# Clinical and radiologic outcomes of biportal endoscopic lumbar discectomy in obese patients: a retrospective case-control study

**DOI:** 10.1186/s12891-022-06082-2

**Published:** 2022-12-22

**Authors:** Hyun-Jin Park, Jun-Young Choi, Ki-Han You, Min-Seok Kang, Woo-Myung Lee, Jin-Tak Hyun, Sang-Min Park

**Affiliations:** 1 Department of Orthopedic Surgery, Spine Center, Hallym University College of Medicine, Kangnam Sacred Heart Hospital, Seoul, South Korea; 2 Department of Orthopedic Surgery, Seoul National University College of Medicine, Seoul National University Bundang Hospital, Seongnam, South Korea; 3 Department of Orthopedic Surgery, Korea University College of Medicine, Anam Hospital, Seoul, South Korea

**Keywords:** Obesity, Body mass index, Lumbar disc herniation, Minimally invasive spine surgery, Biportal endoscopic lumbar discectomy

## Abstract

**Background:**

Obese patients have a higher risk of complications during spinal surgery than non-obese patients**.** To the best of our knowledge, no studies have examined the differences in clinical and radiological outcomes after biportal endoscopic lumbar discectomy (BELD) between obese and non-obese patients. The study evaluated the association between obesity and outcomes after BELD in patients with lumbar disc herniation.

**Methods:**

This was a retrospective case-control study conducted from March 2017 to March 2021 at two hospitals with 360 patients who underwent BELD after showing no improvement with conservative treatment. Clinical and radiologic outcomes were retrospectively analyzed after BELD in the non-obese (body mass index [BMI] < 30 kg/m^2^) and obese (BMI ≥ 30 kg/m^2^) groups. Demographic data and surgery-related factors were compared between the two groups. Clinical outcomes were followed up for 12 months after surgery and analyzed for differences.

**Results:**

A total of 211 patients were enrolled in this study, and through case-control matching, the data of 115 patients (29, obese group; 86, non-obese group) were analyzed. The two groups showed no significant differences in Oswestry Disability Index, European Quality of Life-5 Dimensions (EQ-5D), and visual analog scale scores measured immediately after BELD and 12 months after surgery*.* After surgery, back pain, radiating leg pain, and EQ-5D scores improved. However, there was no significant difference in improvement, residual herniated disc, hematoma, or recurrence between the groups.

**Conclusions:**

Obese patients who underwent BELD for lumbar disc herniation showed no significant difference in clinical and radiologic outcomes compared with non-obese patients.

## Background

Obesity is a known risk factor for low back pain. As body weight increases, excessive load is placed on the intervertebral discs, leading to an abnormal inflammatory response [1–7]. Low back pain is a result of degenerative changes to various mechanisms, including HIVD or sacroiliitis. Obese patients’ back pain might be difficult to diagnose with a conventional physical examination, which has an adverse influence on the patient’s day-to-day activities [8]. In most cases of HIVD, pain and clinical course improve with conservative treatment. Some studies have shown that conservative back pain treatments such medication therapy, physical therapy, and shockwave are beneficial [9]. However, compared with non-obese patients, patients with obesity do not respond well to conservative treatment and are more likely to undergo surgery [10–14].

Patients with obesity are reported to have a higher risk of complications such as surgical site infections or blood loss during spinal surgery than non-obese patients [14–16]. A study of microscopic discectomy performed in patients with obesity and HIVD found no improvement in Oswestry Disability Index (ODI) scores after surgery, but the degree of improvement decreased as the body mass index (BMI) increased. In addition, the obesity group showed long operative times and hospital stays and frequent postoperative complications. There were also no significant differences in lower back pain and radiating pain after surgery [17]. The postoperative outcomes of minimally invasive spinal surgery (MISS) were poorer in obese patients than in non-obese patients.

Recently, a minimally invasive approach to lesion and disc removal has been widely used as surgical treatment for HIVD [18, 19]. Of the MISS options available, biportal endoscopic spine surgery (BESS) is an emerging technique that has been successfully used for posterior lumbar decompression [20]. Compared with microscopic surgery, BESS results in better pain reduction and faster rehabilitation in the early period after surgery; it also has the advantages of reduced scarring and muscle damage [21–23].

In patients with obesity, the space between the skin and surgical field is wide. Therefore, using a microscope to approach the surgical field and handling the surgical instruments within the field is challenging. However, it is possible to overcome these limitations with BESS because the surgical field is larger, which allows freer movement of the surgical instruments [24]. Because of the differences between these two surgical methods, we believe that biportal endoscopic lumbar discectomy (BELD) in patients with obesity can achieve different results from discectomy using conventional microscopic techniques. To the best of our knowledge, there have been no studies on the differences in clinical and radiological outcomes after BELD in patients with and without obesity. Therefore, we aimed to determine the efficiency of discectomy using the biportal endoscopic technique in patients with obesity through a multicenter retrospective study.

## Methods

### Study populations

This retrospective multicenter study was conducted from March 2017 to March 2021 at two hospitals. The orthopedic surgeons at both hospitals have more than 12 years of orthopedic surgery experience, as well as more than 8 years of spine surgery experience. We identified 360 patients who underwent BELD and had shown no improvement with conservative treatment for 2 months. Inclusion criteria of this study as follows: (1) aged > 18 years with low back pain, radiating leg pain (2) symptoms associated with disc herniation seen on magnetic resonance imaging (MRI) (3) disc herniation at single lumbar level. And excluded criteria: (1) patients who underwent reoperation at the lumbar level (2) underwent surgery at multiple levels (3) follow-up period < 1 year after surgery (4) underwent surgery other than BELD. The study enrolled 211 patients, and a retrospective analysis was conducted. All study participants provided informed consent, and the study design was approved by the appropriate ethics review boards.

### Outcome measures

All patients were admitted to the hospital the day before surgery, and baseline demographic data were collected using a standardized form during the preoperative hospitalization period. Height and weight were measured as demographic data, and the patient’s medical, smoking, and surgical history were obtained. According to the definition of BMI (weight in kilograms divided by height in meters squared per the National Institutes of Health criteria), patients with a BMI of > 30 kg/m^2^ were classified as obese, and those with a BMI < 30 kg/m^2^ were classified as non-obese [25]. Patient-reported outcome (PRO) data were measured for clinical outcomes and compared between the two groups. Data regarding the ODI, which consists of items assessing limitation in activities of daily living, and European Quality of Life-5 Dimensions (EQ-5D), which is an evaluation scale for health-related quality of life, were collected before surgery. Low back pain and radiating leg pain were assessed using the visual analog scale (VAS). Patients visited the outpatient clinic periodically after surgery, and PRO data were collected at outpatient visits 3, 6, and 12 months after surgery. Additionally, operative time, postoperative drainage, and length of hospital stay were checked and compared between the two groups as factors related to surgery. To check for postoperative muscle damage, the serum creatine phosphokinase (CPK) ratio (postoperative day 1 CPK/preoperative CPK) was calculated [26]. Immediately after surgery, MRI was used to confirm the removal of the herniated disc and the presence of hematoma or facet joint injury. The MRI images were reviewed by two orthopedic surgeons (with 5 and 10 years of experience, respectively). In addition, the postoperative complications of the patients were analyzed, as well as recurrence and reoperation after surgery were assessed.

### Surgical protocol

**Fig. 1:**
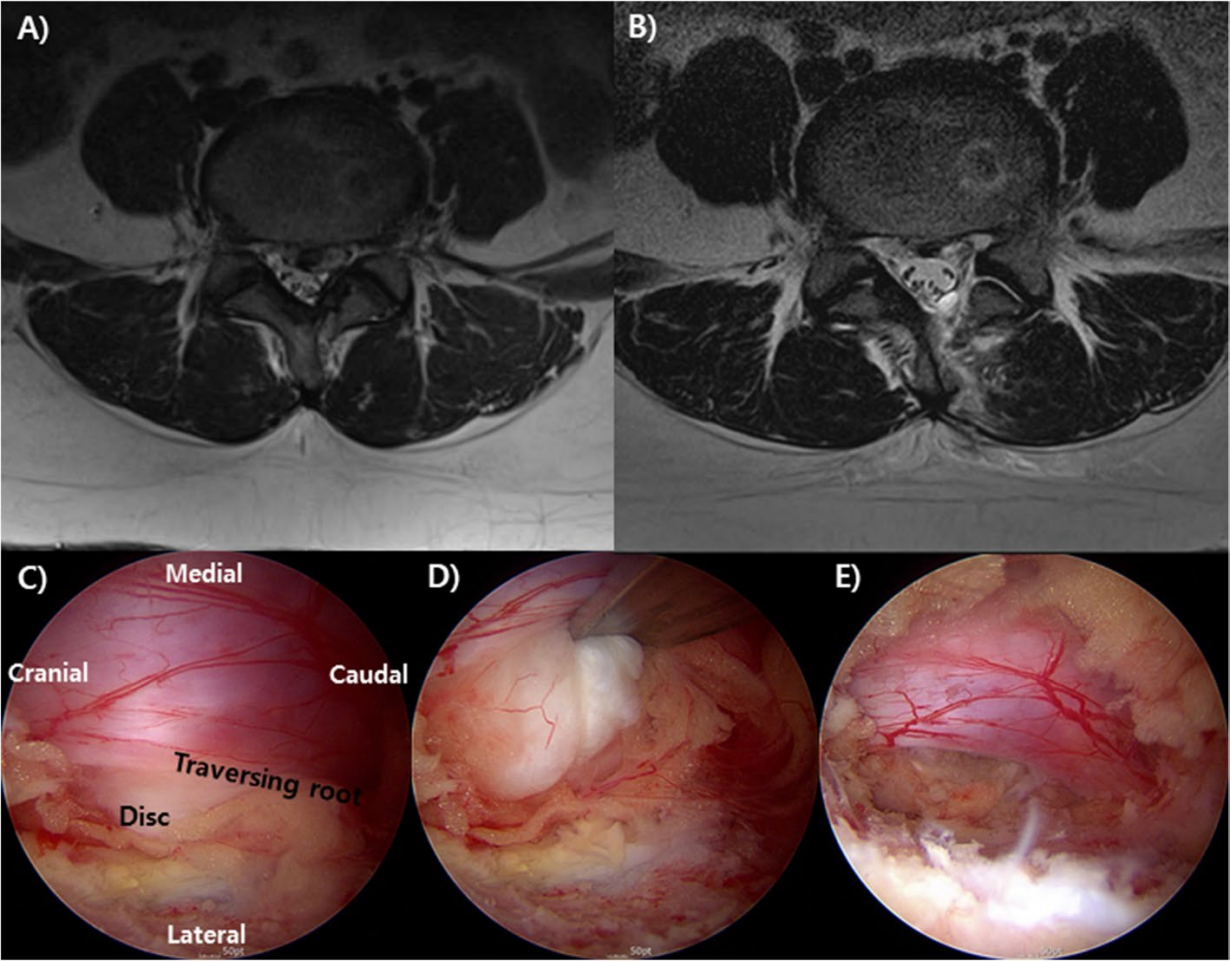
A 32-year-old woman with severe lower back and left lower extremity radiating pain. The patient’s body mass index was 37.8 kg/m^2^. **A** The preoperative T2 axial magnetic resonance image (MRI) shows a huge disc compressing the thecal sac and nerve root. **B** The postoperative T2 axial MRI shows the well-decompressed thecal sac and nerve root. Intraoperative endoscopic findings **(C)** before discectomy, **(D)** during discectomy, and **(E)** after discectomy. Huge pieces of disc material were removed, and the thecal sac and nerve root were freely movable after discectomy

All surgical procedures were performed under general anesthesia. After successful initiation of anesthesia, the patient was placed in the prone position. Before surgery, the surgical site was checked and marked using a portable radiography machine, and a portal was created for biportal endoscopy. Two portals, a viewing portal and a working portal, were used for surgery. The approach was determined based on where the herniated disc was located. After performing laminotomy at the surgical level, the ligamentum flavum was removed and the dura was exposed. Subsequently, the dura was slightly tilted to the side and the herniated disc was removed (Fig. 1). Thereafter, the drain system (BAROVAC®, MayMadi, South Korea) was placed in the operative field, and the operation was terminated.

### Postoperative protocol

From the day after surgery, the patients were allowed to ambulate with orthoses and perform daily activities, except those involving excessive flexion and extension. The drain system was routinely removed on the second day after surgery. Automatic intravenous patient-controlled analgesia was used to control postoperative pain at the surgical site. If the pain at the surgical site had decreased (VAS score ≤ 4) and there were no acute complications after surgery, the patient was discharged.

### Statistical analysis

All statistical analyses were performed using Stata-MP2 version 17.0 (StataCorp LP, College Station, TX, USA). For case-control matching, we used a caliper and the exact matching technique, without replacement. We used the “calipmatch” module in Stata, and each patient with obesity was matched with three non-obese controls. Matching was performed based on two factors: age (caliper match within 4 years) and sex (exact match).

Clinical characteristics, clinical outcomes, and radiographic outcomes were tested for normality using the Shapiro-Wilk test. Normally distributed continuous variables are presented as mean and standard deviation and were analyzed using Student’s t-test, whereas non-normally distributed variables are presented as median and interquartile range and were analyzed using the Mann–Whitney U test. Categorical variables are presented as number and percentage and were analyzed using Fisher’s exact test. A linear repeated-measures mixed model was used to evaluate serial measurements of secondary clinical outcomes (VAS pain score of the back and lower extremities, ODI score, EQ-5D score). To examine the intervention effects at each follow-up point, we used time as a categorical variable (3, 6, or 12 months) and added intervention-time interactions. Using a linear repeated-measures mixed model, we examined inter-group differences over a 12-month period, adjusting for baseline and follow-up time points as categorical variables. When missing values in clinical outcomes existed, we used “last observation carried forward” or “last observation carried backwards” for imputation. Statistical significance was defined as a two-sided *p*-value of < 0.05.

## Results

**Fig. 2:**
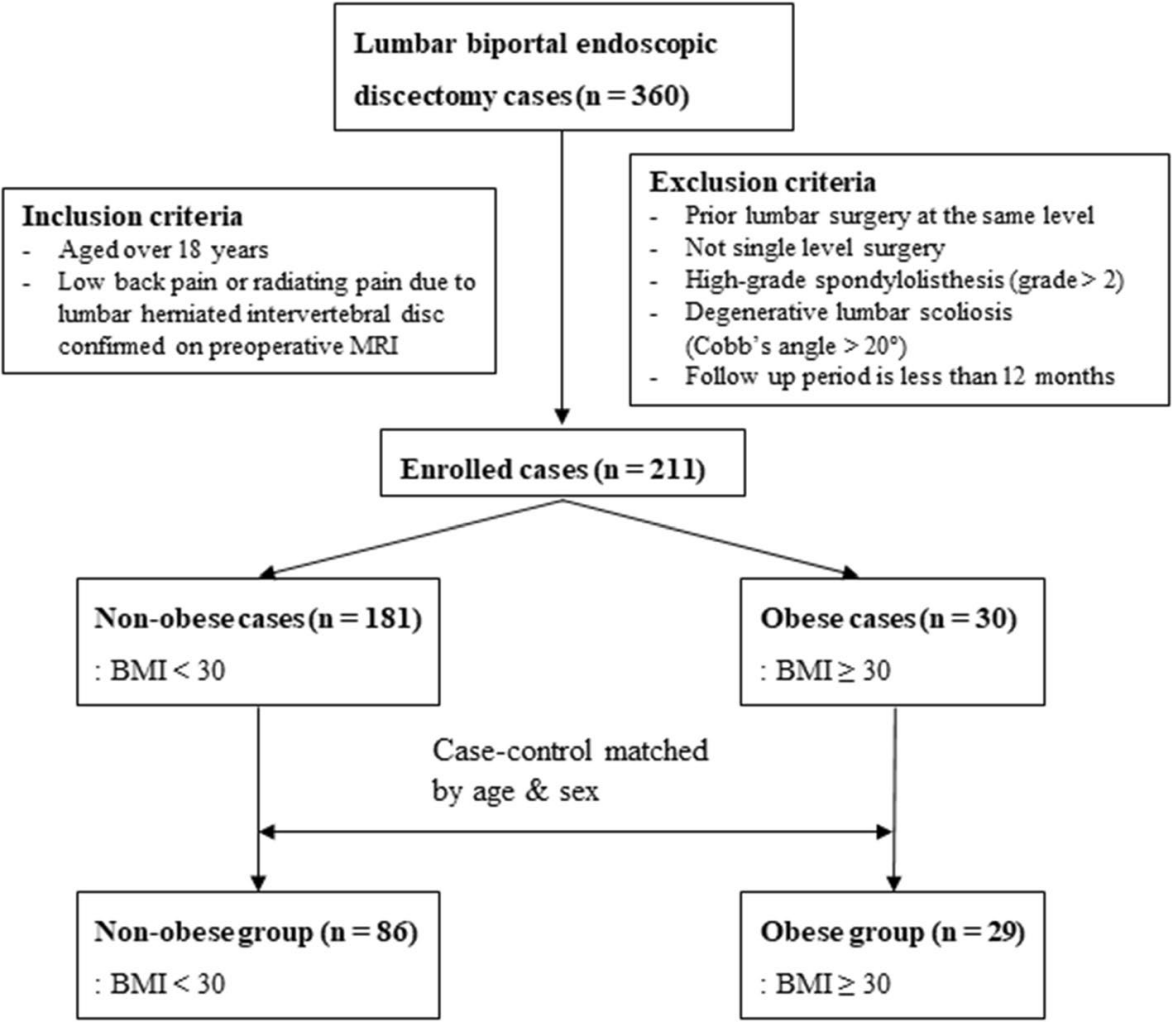
Flowchart of case-control matching process and analysis. A total of 115 matched patients who had undergone biportal endoscopic lumbar discectomy were enrolled. BMI, body mass index; MRI, magnetic resonance image

In this study, 211 patients were enrolled, and the data of 115 patients (29 patients in the obese group and 86 in the non-obese group) were analyzed after case-control matching for age and sex. Before matching, there was a significant difference in age between the two groups, but there was no difference between the groups in most demographics after matching (Fig. 2) (Table 1).

**Table 1:** Baseline patient characteristics according to the presence of obesity before and after case-control matching

Characteristic	Before matching			After matching		
	Non-obese (*n* = 181)	Obese (*n* = 30)	*p*-value	Non-obese (*n* = 86)	Obese (*n* = 29)	*p*-value
Age (years)	53.5 ± 15.0	43.3 ± 16.5	< 0.001	46.6 ± 15.5	44.1 ± 16.2	0.45
Male/Female (%)	98/83 (54.1/45.9)	15/15 (50.0/50.0)	0.67	41/45 (47.7/52.3)	14/15 (48.3/51.7)	0.96
BMI (kg/m^2^)	24.2 ± 2.7	33.0 ± 3.2	< 0.001	24.3 ± 2.7	33.0 ± 3.3	< 0.001
CCI score	0.37 ± 0.74	0.57 ± 1.45	0.26	0.29 ± 0.68	0.59 ± 1.48	0.15
ASA score (%)			0.58			0.51
1	47 (26.1)	6 (20.0)		27 (31.4)	6 (20.7)	
2	105 (58.3)	22 (73.3)		50 (58.1)	21 (72.4)	
3	25 (13.9)	2 (6.7)		7 (8.1)	2 (6.9)	
4	1 (0.6)	0 (0.0)		0 (0.0)	0 (0.0)	
5	2 (1.1)	0 (0.0%)		2 (2.3)	0 (0.0)	
Smoking status			0.53			0.68
Non-smoker	134 (74.0)	20 (66.7)		63 (73.3)	19 (65.5)	
Ex-smoker	18 (9.9)	5 (16.7)		10 (11.6)	5 (17.2)	
Current smoker	29 (16.0)	5 (16.7)		13 (15.1)	5 (17.2)	
VAS for back pain	4.91 ± 2.76	5.06 ± 2.66	0.83	5.06 ± 2.88	5.06 ± 2.66	> 0.99
VAS for leg pain	6.87 ± 2.40	6.71 ± 2.42	0.80	7.04 ± 2.33	6.71 ± 2.42	0.61
ODI	53.2 ± 21.2	50.5 ± 13.2	0.62	54.9 ± 21.0	50.5 ± 13.2	0.43
EQ-5D	0.438 ± 0.200	0.340 ± 0.150	0.21	0.439 ± 0.200	0.340 ± 0.150	0.22
HIVD type			0.62			0.72
Protrusion	7 (3.9)	0 (0.0)		3 (3.5)	0 (0.0)	
Extrusion	123 (68.0)	23 (76.7)		60 (69.8)	22 (75.9)	
Sequestration	5 (2.8)	1 (3.3)		2 (2.3)	1 (3.4)	
Migration	46 (25.4)	6 (20.0)		21 (24.4)	6 (20.7)	
HIVD containment	93 (51.4)	11 (36.7)	0.14	44 (51.2)	10 (34.5)	0.12
HIVD canal compromise			0.09			0.29
Mild (less than 1/3)	76 (42.0)	7 (23.3)		28 (32.6)	7 (24.1)	
Moderate	57 (31.5)	10 (33.3)		33 (38.4)	9 (31.0)	
Severe (over 2/3)	48 (26.5)	13 (43.3)		25 (29.1)	13 (44.8)	
HIVD zone			0.35			0.51
Central	28 (15.5)	7 (23.3)		19 (22.1)	7 (24.1)	
Paracentral	130 (71.8)	19 (63.3)		58 (67.4)	18 (62.1)	
Foraminal	15 (8.3)	4 (13.3)		6 (7.0)	4 (13.8)	
Extraforaminal	8 (4.4)	0 (0.0)		3 (3.5)	0 (0.0)	
HIVD location			0.19			0.19
Disc level	96 (53.0)	22 (73.3)		43 (50.0)	21 (72.4)	
Infrapedicle level	24 (13.3)	3 (10.0)		13 (15.1)	3 (10.3)	
Pedicle level	27 (14.9)	3 (10.0)		14 (16.3)	3 (10.3)	
Suprapedicle level	34 (18.8)	2 (6.7)		16 (18.6)	2 (6.9)	
HIVD calcification	22 (12.2)	5 (16.7)	0.49	6 (7.0)	5 (17.2)	0.10
HIVD side			0.80			0.55
Right	89 (49.2)	14 (46.7)		36 (41.9)	14 (48.3)	
Left	92 (50.8)	16 (53.3)		50 (58.1)	15 (51.7)	
Operation level			0.81			0.87
L2–3	5 (2.8)	1 (3.3)		1 (1.2)	1 (3.4)	
L3–4	23 (12.7)	2 (6.7)		5 (5.8)	2 (6.9)	
L4–5	99 (54.7)	18 (60.0)		52 (60.5)	17 (58.6)	
L5–S1	54 (29.8)	9 (30.0)		28 (32.6)	9 (31.0)	

Data are presented as mean ± standard deviation or as number of patients (percentages). *BMI* body mass index, *CCI* Charlson Comorbidity Index, *ASA* American Society of Anesthesiologists, *VAS* visual analog scale, *ODI* Oswestry Disability Index, *EQ-5D* European Quality of Life-5 Dimensions, *HIVD* herniated intervertebral disc

### Clinical outcomes

**Fig. 3:**
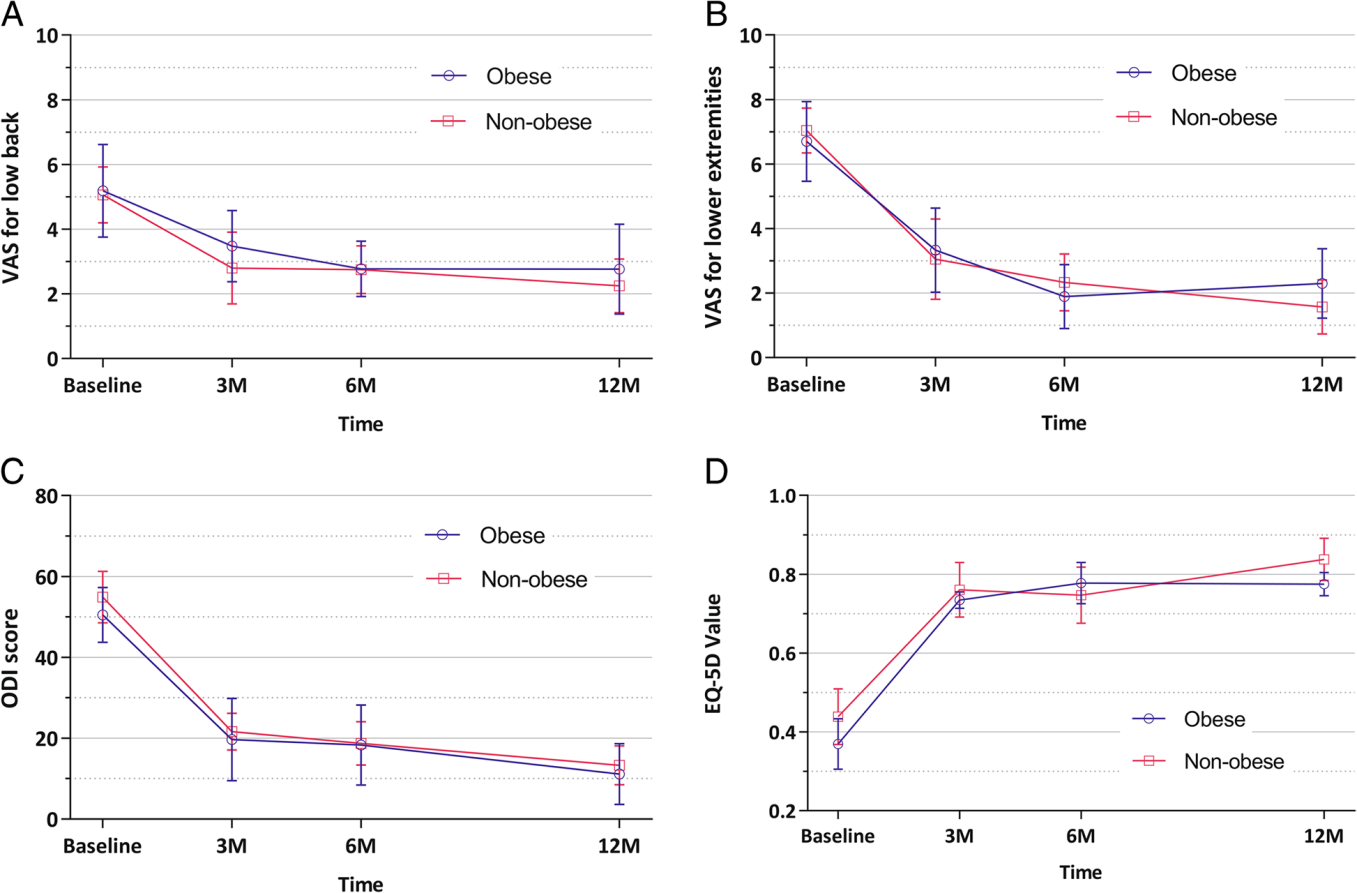
Clinical outcomes between the two groups. VAS, visual analog scale; ODI, Oswestry Disability Index; EQ-5D, European Quality of Life-5 Dimensions

The PRO data (ODI score, EQ-5D value, VAS for low back pain and radiating leg pain) of each group significantly decreased with time up to 12 months after surgery, but this was not significantly different between the two groups. There was no significant difference between the groups in surgery-related factors (operation time, drain, hospital stay, postoperative CPK, and CPK ratio) (Tables 2 and 3) (Fig. 3).

**Table 2:** Clinical outcomes during the follow-up period

Variables	Non-obese (*n* = 86)	Obese (*n* = 29)	Mean difference (95% CI)	*p*-value
**VAS pain score for lower back**				
3 months	3.47 ± 2.30	3.78 ± 2.44	−0.67 (−2.64 to 1.30)	0.513
6 months	2.68 ± 1.82	2.80 ± 1.55	−0.12 (−1.44 to 1.20)	0.783
12 months	2.25 ± 2.14	2.25 ± 2.55	0.00 (−1.82 to 1.82)	0.945
Overall intervention effect^*^	NA	NA	NA	0.967
**VAS pain score for the lower extremities**				
3 months	3.05 ± 2.67	3.11 ± 2.87	− 0.06 (−2.38 to 2.26)	0.891
6 months	2.28 ± 2.16	1.90 ± 1.97	0.38 (−1.20 to 1.97)	0.921
12 months	1.57 ± 2.17	2.63 ± 2.30	− 1.05 (− 2.88 to 0.77)	0.232
Overall intervention effect^*^	NA	NA	NA	0.611
**ODI score**				
3 months	21.65 ± 9.65	19.67 ± 10.44	1.98 (−9.58 to 13.55)	0.860
6 months	18.29 ± 13.25	18.32 ± 13.89	−0.03 (−10.11 to 10.06)	0.742
12 months	13.32 ± 12.43	11.05 ± 8.09	2.27 (−7.85 to 12.38)	0.897
Overall intervention effect^*^	NA	NA	NA	0.884
**EQ-5D value**				
3 months	0.761 ± 0.115	0.735 ± 0.129	0.026 (−0.156 to 0.208)	0.823
6 months	0.756 ± 0.145	0.755 ± 0.104	0.001 (−0.132 to 0.134)	0.990
12 months	0.838 ± 0.128	0.760 ± 0.079	0.078 (− 0.034 to 0.191)	0.264
Overall intervention effect^*^	NA	NA	NA	0.753

*VAS* visual analog scale, *NA* not available, *ODI* Oswestry Disability Index, *EQ-5D* European Quality of Life-5 Dimensions. Data are presented as mean ± standard deviation, unless otherwise indicated. ^*^p-value is from linear mixed models for repeated-measures comparing between interventions during the 12-month follow-up period

**Table 3:** Additional outcomes

Characteristic	Non-obese (*n* = 86)	Obese (*n* = 29)	***p***-value
Operative time (minutes)	74.0 ± 30.2	81.7 ± 29.9	0.23
Length of hospital stay (days)	6.1 ± 4.0	5.0 ± 2.0	0.19
Drainage (mL)	69.7 ± 108.2	78.8 ± 93.6	0.69
Postoperative day 1 CPK	181.23 ± 193.01	204.88 ± 164.75	0.58
CPK ratio^*^	1.54 ± 1.17	1.32 ± 0.56	0.36
Complications during surgery^†^			
Incidental durotomy	2 (2.3)	2 (6.9)	0.245
Facet injury	3 (3.5)	0 (0.0)	0.308
Surgery on the wrong level	0 (0.0)	1 (3.4)	0.084
Complications during follow-up^§^			
Asymptomatic hematoma	13 (15.1)	2 (6.9)	0.256
Residual herniated disc	41 (47.7)	13 (44.8)	0.791
Surgical site infection	0 (0.0)	0 (0.0)	0.416
Recurrent disc herniation resulting in reoperation	5 (5.8)	0 (0.0)	0.184
Recurrent disc herniation not requiring reoperation	10 (11.6)	6 (20.7)	0.223

Continuous data are presented as mean ± standard deviation. Categorical data are presented as number of patients (percentages). ^*^CPK ratio = postoperative day 1 CPK/preoperative CPK. ^†^Complications that occurred during surgery that were confirmed during or immediately after surgery. ^§^Complications not identified until the final follow-up after surgery. *CPK* creatine phosphokinase

### Radiographic outcomes

No patients in the obese group and three patients in the non-obese group had facet joint injury on MRI immediately after surgery. Asymptomatic hematomas were observed in two patients in the obese group and 13 patients in the non-obese group. Residual herniated disc was observed in 13 patients in the obese group and 41 patients in the non-obese group (Table 3). There was no significant difference in MRI findings after surgery between the groups.

### Complications

Two patients each in the obese and non-obese groups were treated for complications (incidental durotomy and hematoma) within 30 days of surgery. 30 days after surgery, six patients in the obese group and 15 patients in the non-obese group were treated nonoperatively for disc recurrence (via medication or lumbar injection); however, five patients in the non-obese group eventually underwent reoperation. There were no significant differences in postoperative complications or recurrence between the two groups (Table 3). Major complications such as neurological injury, pulmonary thromboembolism, cardiovascular events, cerebrovascular events, surgical site infection, and surgery-related death were not observed.

## Discussion

This study was a retrospective, multicenter study that aimed to determine whether the clinical and radiological outcomes of patients with obesity after BELD are different compared with those of non-obese patients. BELD can be considered a relatively useful surgical method in patients with obesity because there is no difference in the improvement of the patient’s clinical picture, radiological results, and complications such as recurrence and reoperation.

It is currently controversial whether patients with obesity have better outcomes after lumbar surgery than patients without. According to previous studies, patients with obesity had worse outcomes in terms of surgery-related factors such as excess blood loss, length of hospital stay, operative time, and surgical site infection as a result of lumbar MISS [27]. In a study on lumbar interbody fusion with MISS, the obese group showed more complications and poorer results, such as longer hospital stays, than the non-obese group [28]. While patients with obesity reported poor clinical outcomes after undergoing lumbar MISS, according to some studies, there was no difference in clinical results between patients with and without obesity after microscopic discectomy [17]. And for disc herniation, there is a study that gender is not related to the success of conservative treatment or surgical treatment, and the occurrence of recurrent disc herniation after surgery [29–31]. Therefore, we designed this study and analyzed the results to determine the clinical and radiological results of BELD in patients with obesity and gender difference with HIVD.

Conducting spinal surgery on patients with obesity is challenging for several reasons: (1) anesthesia is problematic; (2) the abdominal pressure can increase when using a normal Wilson frame to hold the patient in the prone position; therefore, bleeding can occur more often, which has been reported to be related to the occurrence of wound problems or surgical site infections [15, 16]; and (3) it is difficult to access the surgical field during open surgery and the field of view is limited. In particular, the working space of the tubular retractor used in the microscopic technique becomes smaller, and it is more difficult to maneuver it when the length of the tube is increased. However, because there is a high degree of freedom in moving the instrument in BESS, the difficulty of the operation is not substantially increased. Therefore, biportal endoscopy is considered more suitable than microscopy when performing spinal surgery on patients with obesity with HIVD.

**Fig. 4:**
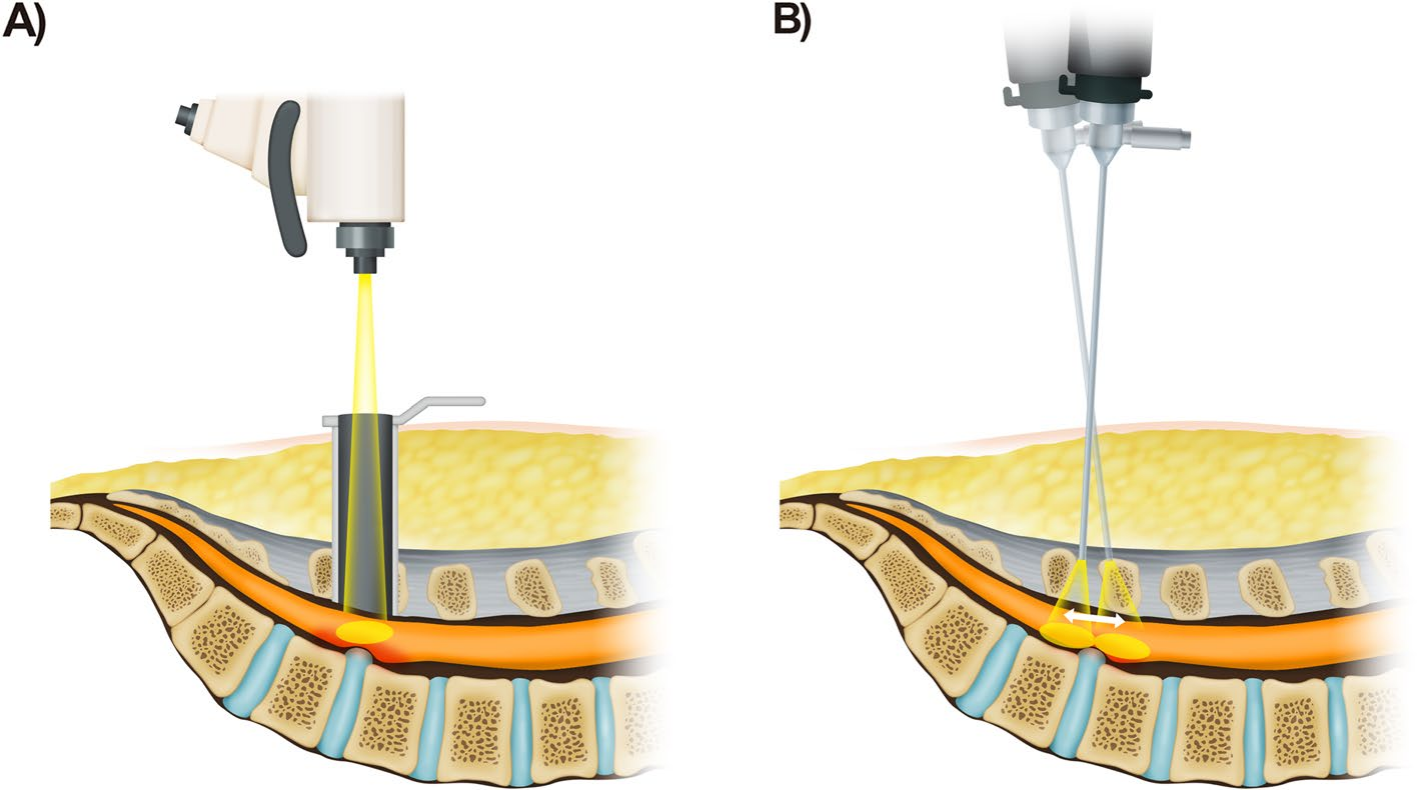
Microscopic surgery using a tubular retractor **(A)** versus biportal endoscopic surgery **(B)** in an obese patient. In patients with obesity, the surgical field is deeper and muscle retraction is more difficult. In such cases, biportal endoscopic surgery can be performed with fewer restrictions

BESS reduces the size of the incision and because constant pressure is generated by the continuous water irrigation during surgery in this technique, intraoperative bleeding and the infection rate can be reduced [32]. Patients with obesity require a more notable incision and have greater muscle depth than patients without obesity, making the use of electrocautery inevitable. However, because BESS uses radiofrequency and not electrocautery, less muscle injury is expected (Fig. 4) [33].

Our study found no difference in the clinical and radiological results between the obese and non-obese groups after BELD. This may result from the advantages that BELD offers regarding a better surgical field of vision and less muscle injury. Previous studies have shown that obese people are more likely to undergo infection and excessive bleeding after spine surgery [14–16]. However, in our study results, we confirmed that there was no difference in the amount of bleeding and the infection rates between obese and non-obese groups. Through this, it is possible to know the advantages of the BELD compared to the conventional surgical techniques. In addition, in a study on microscopic discectomy surgery, ODI scores also showed a tendency to decrease as BMI increased [17]. In this study, it was confirmed that there was no difference in ODI scores after surgery between the obese and non-obese groups. This suggests that using an endoscope rather than a microscope in the obese disc herniation patient’s surgery could lower the degree of disability in patients after the surgery. Of course, a well-designed study is needed for this. Therefore, BELD is considered a good option for spinal surgery in patients with obesity.

In demographics, 1:1 age and sex matched case control studies were conducted to reduce bias. In this study, no study results were obtained according to age or gender. In a follow-up study, it would be useful to stratify age and compare surgical outcomes according to gender.

This study has some limitations. First, it was retrospective and involved a small number of patients. BELD is a relatively recent surgical development, and thus few patients with obesity were available for this study [20, 34]. To overcome this limitation, we recruited patients from two institutions and reduced selection bias through case-control matching. Second, the postoperative follow-up period was relatively short (12 months). Although clinical outcomes and recurrence that may change after 12 months have not been studied, the results up to 12 months seemed similar to those of the Spine Patient Outcomes Research Trial study, which followed up similar patients for 3 years. Therefore, it is thought that the subsequent course will show similar results. Although clinical outcomes after several years are important, the clinical outcomes at 12 months after surgery are also valuable; as such, the current study can be clinically meaningful. Third, we used PROs to evaluate clinical outcomes to obese and non-obese patients in our study. These measure the subjective degree of pain, disability, and quality of life of patients. According to a study, patients with obesity performed more self-assessments than those without obesity and showed a tendency toward negative self-assessment [35]. Therefore, differences may have already occurred during the data collection process in obese and non-obese patients. To resolve this difference, while the patient was completing the questionnaire, the investigator objectively explained the questions to allow the patient to select an appropriate score for the symptoms and discomfort they currently felt.

The long follow-up period more than 1 year after surgery in a study designed as a randomized controlled trial and investigating various risk factors that can affect the outcome of surgery is thought to be able to determine the results of BELD surgery more accurately in obese patients in the follow-up study.

## Conclusions

To the best of our knowledge, this is the only study to specifically compare the clinical and radiographic outcomes of patients with and without obesity after BELD. It revealed that the clinical and radiographic outcomes and complications in obese patients were not significantly different from those in non-obese patients. Therefore, BELD is a feasible surgical procedure for obese patients with HIVD who are indicated for lumbar discectomy.

## Data Availability

The datasets used and analyzed during the current study available from the corresponding author on reasonable request.

## Abbreviations

BELD: Biportal endoscopic lumbar discectomy
BESS: Biportal endoscopic spine surgery
BMI: Body mass index
CPK: Creatine phosphokinase
EQ-5D: European Quality of Life-5 Dimensions
HIVD: Herniated intervertebral disc
MISS: Minimally invasive spinal surgery
MRI: Magnetic resonance imaging
PRO: Patient-reported outcome
ODI: Oswestry Disability Index
VAS: Visual analog scale
